# Data regarding talent management practices and innovation performance of academic staff in a technology-driven private university

**DOI:** 10.1016/j.dib.2018.05.081

**Published:** 2018-05-26

**Authors:** Odunayo Salau, Adewale Osibanjo, Anthonia Adeniji, Olumuyiwa Oludayo, Hezekiah Falola, Ebeguki Igbinoba, Opeyemi Ogueyungbo

**Affiliations:** Covenant University, Nigeria

**Keywords:** Talent, Management, Development, Retention, Attraction, Innovation, Performance

## Abstract

The article presented an integrated data on talent management practices and innovation performance of academic staff in a technology-driven private university in Nigeria. The study adopted a quantitative approach with a survey research design to establish the major determinants of talent management practices. The population of this study included academic staff and the use of questionnaire was adopted to elicit from the study population. Data was analysed with the use of structural equation modelling and the field data set is made widely accessible to enable critical or a more comprehensive investigation. The findings identified talent development and retention strategies as predictors for facilitating innovation performance in the sample University. It was recommended that management of the sampled university will consistently need to adopt reliable range of strategies to attract and retain people for excellence performance.

## Specification Table

**Table:** 

**Subject area**	Business, Management
**More Specific Subject Area:**	Organizational Behaviour and SHRM
**Type of Data**	Primary data
**How Data was Acquired**	Through questionnaire
**Data format**	Raw, analyzed, Inferential statistical data
**Experimental Factors**	Population consisted of selected academic staff of a technology-driven university in Nigeria. The researcher-made questionnaire which contained data on talent management practices and Innovation performance
**Experimental features**	Talent management is a major determinant of organisational success and should be at the forefront of organisational policies and culture.
**Data Source Location**	Lagos, Nigeria
**Data Accessibility**	Data is included in this article

## Value of data

● The data can be used by managers to properly make decisions that in the long-run would lead to goal attainment in the organization.
● The data can be used to enlighten managers on the importance of retention attributes and how it can be beneficial to the overall wellbeing of the organization.
● The data provides ample knowledge on how different organisational retention attributes can interact effectively by building healthy relationship and sustaining greater commitment.
● Generally, data acquired from this study would be significant for organizational goal achievement, proper building of corporate image which would in turn lead to organizational success
● The data described in this article is made widely accessible to facilitate critical or extended analysis.

## 1 Introduction

Business firms tend to improve performance through generation and implementation of various policies, strategies and actions that would help to retain committed employees. Talent management is a major determinant of organisational success but highly misconstrued in a competitive and demanding environment like the educational sector. Today, institutions especially the private universities in Nigeria are becoming more conscious of how and why talents need to be identified and managed. Several strategies have been put in place by many private universities to stimulate these practices, but despite the efforts, the issue of turnover, brain drift and low performance still become worrisome especially in the Nigeria׳s context.

## 2 Data

**Fig. 1:**
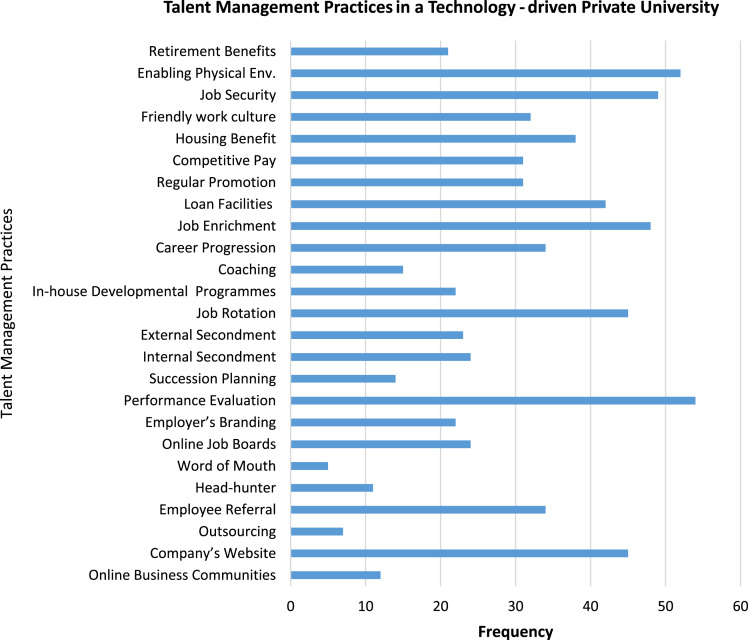
Talent management practices in a technology-driven private university.

The study is quantitative in nature and data were retrieved from staff (teaching and non-teaching) of Covenant University. This paper adopts the talent management measurement proposed by Winfield [9] and it covers the three (3) major indicators such as talent attraction (recruitment); talent development (capacity building) and talent retention. Meanwhile, the measurement for talent attraction (recruitment) is designed by modifying the five questions proposed by Lyria [10]. The measurement for talent development (capacity building) is based on the four questions of Chikumbi [11], with an emphasis on performance evaluation, succession planning, job rotation, coaching, internal and external secondments, etc. Meanwhile, the measurement for talent retention is developed by modifying the questions designed by Poorhosseinzadeh and Subramaniam [7]. In addition, this paper refers to the measurements and indicators proposed by Sofat [8] for innovation performance. The decision to elicit information from the staff was based on the fact that they are often in the best position to describe their real employment relationships and how it has influenced their creative and critical thinking as presented in Fig. 1.

**Fig. 2:**
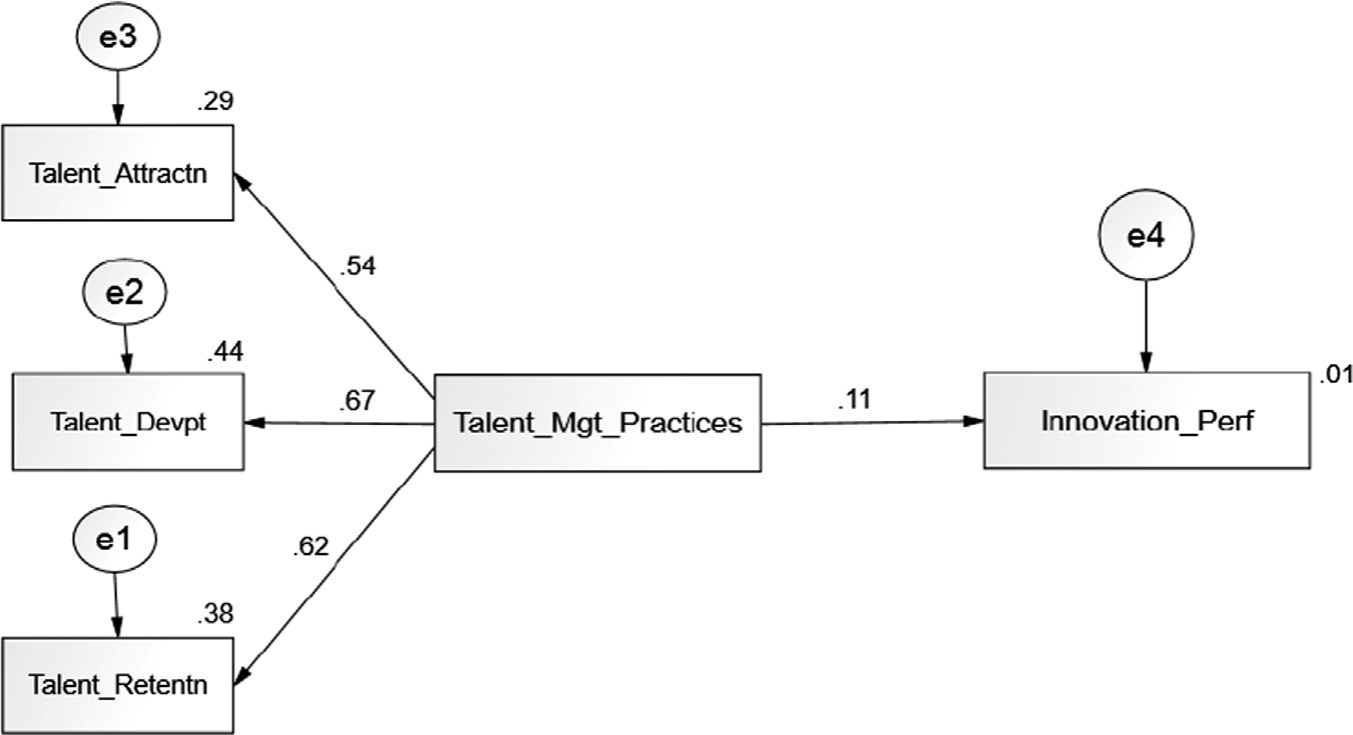
Regression weights of the variables.

After validating the data collected from the survey, we further evaluated the structural relationships in the research model. We first use our data to test the benchmark model of Lee and Choi׳s [18] that has been empirically established. This analysis allows us to verify the integrity of our measurements as compared with existing research. Then, we continue to test the extended HRM practices model as recommended by Anderson and Gerbing [19] to evaluate the structural model as presented in the Fig. 2 and Table 1 respectively.

**Table 1:** The model fit summary showing the goodness of fitness.

Goodness of fit	SEMs value	Recommendation values	Remarks
Chi­Square/Degree of Freedom (CMIN/DF)	2.319	≤3.00	Acceptable fit
Normed Fit Index (NFI)	0.973	≥.90	Good fit
Comparative Fit Index ( CFI)	0.946	≥.90	Very Good fit
Incremental Fit Index (IFI)	0.937	≥.90	Good fit
Root Mean Squared Error of Approximation (RMSEA)	.059	≤.08	Good fit
Goodness of Fit (GFI)	.941	≥.90	Good fit

Results in Table 1 dictate that the value of *χ*^2^ is within the acceptable range of 1 and 3 as suggested by Browne and Cudeck [13] and Hu and Bentler [12]. On top of that, the incremental fit, NFI, TLI, CFI, and GFI were above 0.90 [14,15]. All the basic assumptions were acceptable and prove that the data met the conditions of basic assumption in structural equation modelling.

## 3 Experimental design, materials and methods

An empirical study was conducted to test the proposed research model. Survey instruments were distributed to 350 staff of the leading private university in Africa and has also emerged the winner of the Best Use of Technology Award 2016 among the private universities in Nigeria. The same University among others was awarded the Centenary ICT Driven University of the Year in 2014, the Overall Best University in Nigeria and West Africa and number 15 in Africa on the Webometric Ranking (February 2015) as well as African Private University of the Year (2015), by African Leadership Magazine Group in March, 2016.

All participants were selected based on their substantive amount of work experience with the institution; as such they were able to provide useful information regarding the survey questions. Of those surveyed, only 313 respondents filled out and returned the questionnaire without missing or invalid data. Data were gathered from academic staff and non-academic staff directors, and other categories of employees across the institution with the aid of a researcher- made questionnaire based on the works of [1], [2], [3], [4], [5], [6]. The demographic data presented information based on gender, age, education and experience as well as questions related to talent management practices and innovation performance. This paper constructs its research structure in accordance with the characteristics of the sampled sector, and literature addressing the practices of talent management and innovation performance outcomes.

The collected data were coded and analysed using SPSS version 22. Data was analysed applying descriptive and inferential statistical tests. Importantly, the study participants were selected based on the following inclusion criteria:

**Inclusion criteria**:

• Participants were employees of the sampled technology-driven university.
• Participants signed the consent form provided and have worked with the firm for a minimum period of 2 years.
• Participants were accessible as at the time of the survey and interviews.

This paper adopts the measurement proposed by Ueno [17] and Delery [16] as a reference structure to examine the practices of HRM. This measurement focuses on the evaluation of talent attraction, talent development and talent retention. It covers the process of identifying the strategies for job advert and recruitment; examining the main reasons for participants agreeing to stay with institution; the extent of capacity development programmes; the existence of a clearly specified daily job description; the degree of retention strategies adopted and implemented; and the existence of the desire to change jobs. The section on innovation performance was adapted from a previously validated questionnaire.

Even though emerging issues in HRM have received much attention in the management literature over the last decade, only limited research has established how talent management practices can be used to drive on growth and prosperity for many organisations empirically. Motivated by a critical synthesis of literatures and consistent with prior research, our study shows that talent management practices improve innovation performance, which subsequently results in superior organizational performance. The researchers ensured that respondents were well informed about the background and the purpose of this research and they were kept abreast with the participation process. Respondents were offered the opportunity to stay anonymous and their responses were treated confidentially.
