# Supplementary material for: Data regarding talent management practices and innovation performance of academic staff in a technology-driven private university
Source: Data Brief. 2018 May 26;19:1040–5. doi: 10.1016/j.dib.2018.05.081 (PMC6139485; doi:10.1016/j.dib.2018.05.081)
Supplement: Supplementary file 1 — Supplementary material [file mmc1.docx]

**DECLARATION OF INTEREST FORM**

[**DATA REGARDING TALENT MANAGEMENT PRACTICES AND INNOVATION PERFORMANCE OF ACADEMIC STAFF IN A TECHNOLOGY-DRIVEN PRIVATE UNIVERSITY**](https://www.sciencedirect.com/science/article/pii/S2352340918302816)

Odunayo **SALAU**; Covenant University

[odunayo.salau@covenantuniversity.edu.ng](mailto:odunayo.salau@covenantuniversity.edu.ng)

Adewale OSIBANJO; Covenant University

adewale.osibanjo@covenantuniversity.edu.ng

Anthonia ADENIJI; Covenant University

[anthonia.adeniji@covenantuniversity.edu.ng](mailto:anthonia.adeniji@covenantuniversity.edu.ng)

Olumuyiwa OLUDAYO Covenant University

olumuyiwa.oludayo@covenantuniversity.edu.ng

Hezekiah FALOLA; Covenant University

[hezekiah.falola@covenantuniversity.edu.ng](mailto:hezekiah.falola@covenantuniversity.edu.ng)

Ebeguki IGBINOBA; Covenant University

[ebe.igbonoba@covenantuniversity.edu.ng](mailto:ebe.igbonoba@covenantuniversity.edu.ng)

Opeyemi OGUEYUNGBO; Covenant University

opeyemi.ogueyungbo@covenantuniversity.edu.ng

We, the Authors of paper entitled above certify that we have seen and approved the final version of the manuscript being submitted. This is an original work and has not received prior publication and is not under consideration for publication elsewhere. It is also important to state that there is no financial/personal interest or belief that could affect our objectivity and to prevent ambiguity, we humbly want to state explicitly that there is no conflicts of interest as regards the review and publication of this paper.

Thank you.

SALAU Odunayo Paul

*Signed*
